# Impact of Organ Donor Pretreatment With Anti-Thymocyte Globulin in a Murine Model of Allogenic Kidney Transplantation

**DOI:** 10.3389/ti.2024.13997

**Published:** 2025-01-07

**Authors:** An He, Yiren Yang, Katja Kotsch, Arne Sattler

**Affiliations:** ^1^ Charité–Universitätsmedizin Berlin, Corporate Member of Freie Universität Berlin, Humboldt-Universität zu Berlin, and Berlin Institute of Health, Department for General and Visceral Surgery, Berlin, Germany; ^2^ Department of Urology, The Second Affiliated Hospital, School of Medicine, Zhejiang University, Hangzhou, Zhejiang, China

**Keywords:** anti-thymocyte globulin, kidney transplantation, donor pre-treatment, passenger leukocytes, kidney function

## Abstract

Kidney transplantation is the treatment of choice for end-stage organ failure. To improve transplantation outcomes, particularly of “marginal” organs from extended criteria donors (ECD), attempts have been made to therapeutically modulate donor or graft pre-transplantation. Anti-thymocyte globulin (ATG) has a history as lymphocyte-depleting, immunosuppressive drug for treating rejection episodes post transplantation. In this study, however, we aimed to comprehensively analyze the effects of ATG donor pre-conditioning in a mouse model of kidney transplantation. ATG pre-treatment of potential donors led to a broad depletion of T- and NK cells in peripheral blood, non-lymphoid (including kidney) and lymphoid organs within 48 h, whereas myeloid cells were spared. ATG was also effectively depleting renal innate lymphoid type 1 and 2 cells. Importantly, transplantation of kidneys from ATG pre-treated donors into fully mismatched recipients showed only mild effects on leukocyte re-composition post transplantation. In line with this, serum creatinine and urea levels were similar in animals receiving kidneys from ATG treated donors or controls, demonstrating that donor treatment had no effect on allograft function in the early post-transplantation phase. In summary, our findings are suggestive of a more cell-type-specific depletion strategy in concert with an experimental model better reflecting aspects of clinical transplantation.

## Graphical Abstract

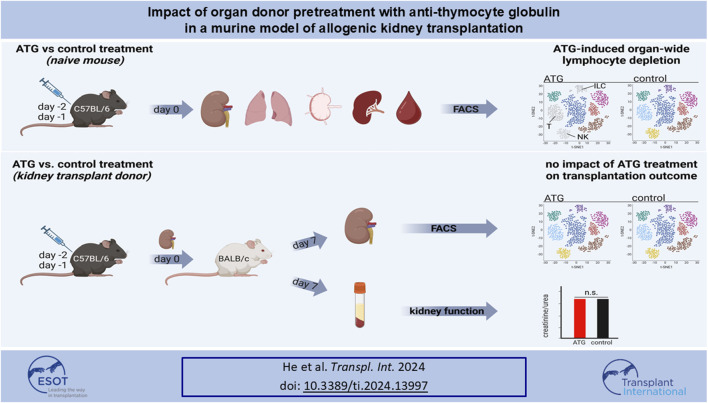

## Introduction

The increasing demand for organ transplantation and the shortage of available organs limit the success of transplantation programs. Consequently, acceptance of expanded criteria donor (ECD) organs, being associated with a higher risk of unfavorable transplantation outcome, has become an increasing reality [1–3]. Among the most prominent characteristics distinguishing ECD from Standard Criteria Donors (SCD) are risk factors such as brain death, prolonged cold ischemic time, increased donor age, hypertension or diabetes. Altogether, these conditions could impact the intra-renal milieu towards a higher inflammatory burden [4], thereby compromising long-term graft function [3]. On the cellular level, age-related changes in donor organ composition have been shown to include functional programming of resident lymphocytes towards a more pro-inflammatory phenotype [5]. Furthermore, it has been meanwhile revealed in a number of studies that such donor cells, being transferred to the organ recipient as “passenger leukocytes” [6], could impact transplantation outcomes in a celltype-specific manner [7–9].

Current experimental and clinical research therefore aims at therapeutically modulating grafts pre-transplantation to improve organ function. Principally, strategies focusing on systemic (deceased) donor pre-treatment or therapeutic targeting of the explanted graft have been proposed (summarized in [10, 11]). Amongst post-explantation procedures, we could demonstrate that perfusion of human kidneys with rabbit anti-human ATG results in improved short-term graft function [12]. Such peri-operative window of intervention is short by nature of the surgical procedure; furthermore, at low temperatures, therapeutics targeting biological processes might not unfold their full potential. Particularly the latter aspect also applies to machine perfusion as a novel framework for graft modification: although meanwhile a standard organ preservation method e.g., in the Netherlands [13], machine perfusion is routinely conducted hypothermically, thereby likely limiting drug metabolism.

On that background, pre-treatment of deceased, brain dead donors, being kept for hours near body temperature pre-explantation, might constitute an alternative option for graft modification. In clinical liver transplantation, methylprednisolone treatment not only reduced systemic inflammation associated with brain death in hepatic donors, but also ameliorated ischemia/reperfusion injury post-transplantation in concert with reduced acute rejection episodes [14]. In line with this, donor treatment with low dose dopamine improved renal function after transplantation in a large randomized controlled trial [10]. So far, anti-thymocyte globulin for pre-conditioning of the donor has been mainly studied in animal models of ischemia/reperfusion injury [15–17]. Its precise effects on leukocyte depletion across organs after donor pre-treatment and ensuing consequences for experimental kidney transplantation remained to be determined.

We therefore comprehensively studied quantitative changes within lymphoid and myeloid cell lineages in multiple murine lymphoid and non-lymphoid organs after intra-peritoneal anti-murine ATG administration. Furthermore, we analyzed its impact on cellular infiltration and renal function in a fully mismatched model of murine kidney transplantation.

## Materials and Methods

### Animals

8–12-week-old male wildtype BALB/c, C57BL/6, and B6.SJL-Ptprca Pepcb/BoyJ (CD45.1^+^ for donor/recipient discrimination) mice were purchased from Charles River Laboratories (Charles River, Cologne, Germany) and kept under standard laboratory animal conditions, receiving human care in compliance with the “Principles of Laboratory Animal Care” prepared by the National Academy of Sciences and published by the National Institutes of Health (NIH Publication No. 86–23, revised 1985). All animal experiments were approved by the Landesamt für Gesundheit und Soziales Berlin, Germany (G 0089/16).

### ATG Treatment

Anti-mouse ATG was prepared by purifying the IgG fraction from serum of rabbits immunized with pooled thymocytes prepared from NOD, C3H/He, DBA/2, and C57BL/6 mice (Sanofi). ATG was administered to C57BL/6 mice intra-peritoneally at 25 mg/kg bodyweight at day −2 and −1. Control animals received purified IgG from unimmunized rabbits. Animals were sacrificed on day 0 for direct cellular analysis or kidney procurement for transplantation.

### Murine Kidney Transplantation

Allogeneic renal transplantations were performed as previously described [5, 18, 19]. Briefly, the left donor kidney was flushed with saline containing heparin (100 U/mL, Panpharma, Trittau, Germany) and procured. End-to-side anastomoses between the renal donor vessels and the recipient’s abdominal aorta and inferior vena cava were performed following a knotless technique. For urinary tract reconstruction, the ureter was directly anastomosed into the bladder. The duration of cold and warm ischemia of allografts was maintained at 30 min, respectively. Animals were sacrificed on day 7 without receiving immunosuppression.

### Assessment of Kidney Function

Serum samples were stored at −20°C until creatinine and urea were measured using the CREP2 Creatinine Plus version 2 and Urea/BUN assays, respectively, on a Roche/Hitachi Cobas C 701/702 system (Roche, Basel, Switzerland).

### Isolation of Mononuclear Cells

For isolating renal mononuclear cells (MNCs), kidney tissue was mechanically dissociated and digested in RPMI medium (Corning, Manassas, VA, United States) supplemented with collagenases II and IV (Gibco/Invitrogen, Worthington) and DNase I (Roche Diagnostics) for 45 min at 37°C. Afterwards, leukocytes were enriched using CD45 Microbeads over MACS LS columns (both Miltenyi Biotec, Bergisch Gladbach, Germany). Mechanically dissociated lung tissue was digested with collagenase II and DNAse I; leukocytes were retrived after red blood cell lysis using ACK buffer. MNCs from spleen, lymph nodes and peripheral blood were isolated by density gradient centrifugation.

### Flow Cytometric Analysis

Typically, 1 × 10^6^ cells were surface stained with the respective antibodies listed in Supplementary Figures S1, S3. For intracellular staining, cells were fixed, permeabilized (FoxP3/transcription factor staining buffer set; Thermo Fisher, Darmstadt, Germany) and stained with the respective anti-transcription factor antibodies (Supplementary Figure S3). Data was acquired on a FACS Fortessa X20 (BD Biosciences, Heidelberg, Germany) and analyzed using FlowJo software 10 (BD Biosciences). A gating strategy for identification of lymphoid and myeloid cells is depicted in Supplementary Figure S2. As the predominant population, CD11b^+^F4/80^+^ macrophages were quantified in kidney, whereas Ly6C^+^MHC-II^-^ monocytes were analyzed in all other organs. Innate lymphoid cells were identified as illustrated in Supplementary Figure S4. For generation of t-Distributed Stochastic Neighbor Embedding (t-SNE) plots, data from samples of interest were concatenated, followed by t-SNE analysis in FlowJo. Graphical illustrations were designed with Biorender.

### Statistics

Statistical analysis was performed using GraphPad Prism 8.4.3 (GraphPad, Boston, MA, United States). Distribution of values was assessed by Kolmogorov-Smirnov normality test. Depending on distribution, comparisons were conducted using either T- or Mann–Whitney U test. Statistical significance was considered for p values ≤0.05.

## Results

### Treatment of the Naïve Organ Donor With Anti-Mouse ATG

**FIGURE 1:**
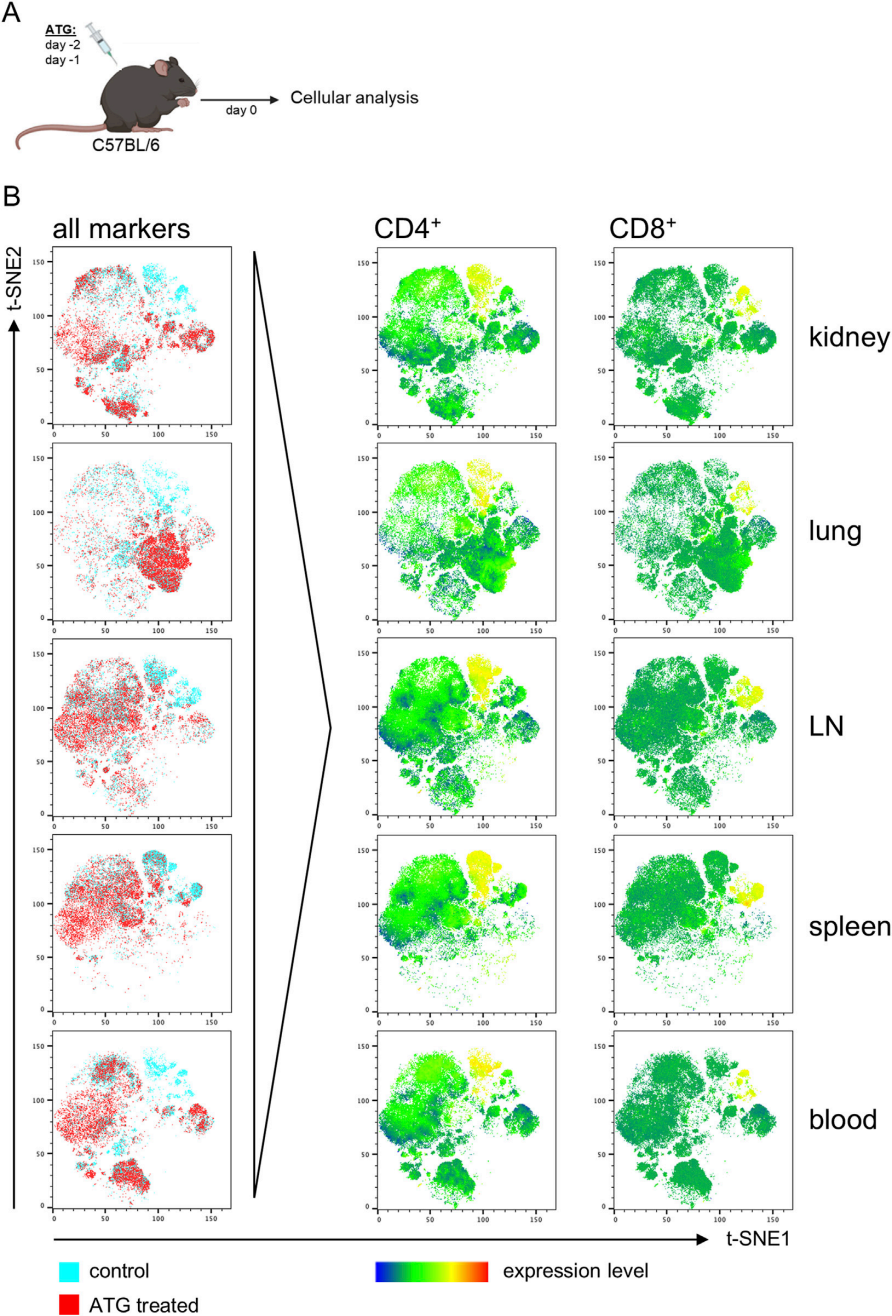
ATG-induced T cell depletion across organs in naive mice. **(A)** Intra-peritoneal ATG treatment scheme of naïve C57BL/6 mice. **(B)** t-SNE plots of flow cytometric data illustrate separate clustering of cells from the indicated organs derived from control vs. ATG-treated animals. For t-SNE analysis, cells were pre-gated on live CD45^+^ leukocytes; thereafter, datasets from n = 4–7 animals/group were concatenated. All remaining markers listed in Supplementary Figure S1 were used for clustering. Data from control (blue) and ATG-treated (red) animals were overlaid to identify differences in cluster composition. For prominently different clusters, cellular identity was determined based on expression of the respective lineage markers, thereby e.g., identifying CD4^+^ and CD8^+^ T cell depletion in ATG-treated animals.

First, we aimed to address how application of ATG to a potential solid organ donor impacts the lymphocyte composition in various organs. Naïve C57/BL6 mice were therefore treated with ATG before organ harvesting as summarized in Figure 1A. Control Ig treated mice of the same age and sex served as controls. For assessing all major lymphoid and myeloid cell subsets, mononuclear cells isolated from kidney, lung, spleen, lymph nodes and peripheral blood were surface stained using a FACS panel covering major cell lineage markers as depicted in Supplementary Figure S1, followed by acquisition on an BD Fortessa X20 flow cytometer.

**FIGURE 2:**
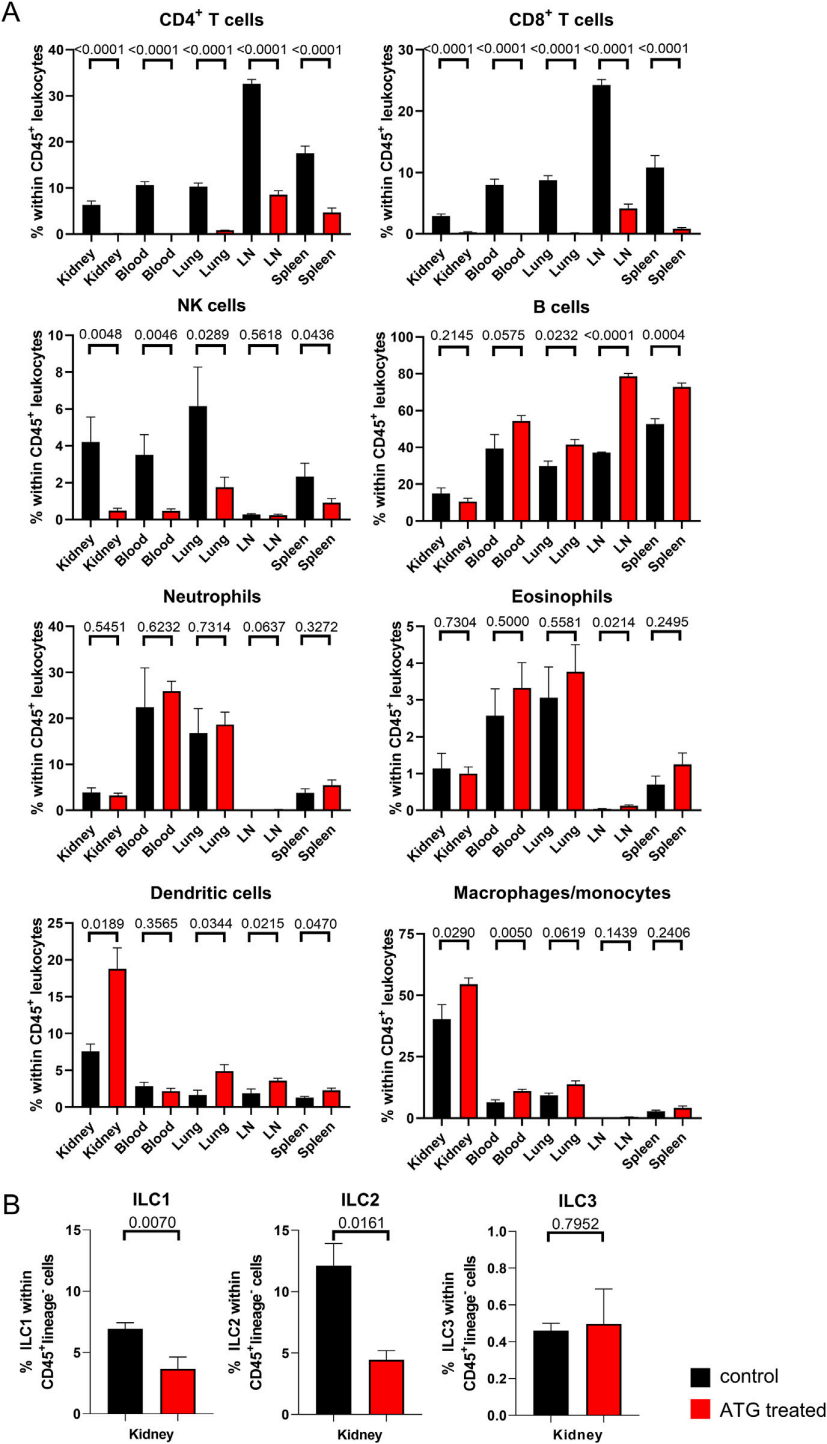
Lymphocyte depletion in naive mice after ATG treatment. **(A)** The indicated leukocyte lineages were quantified by FACS in kidney, blood, lung, lymph node (LN) and spleen in control (con) and ATG-treated (ATG) C57BL/6 animals. **(B)** Renal composition of type 1, 2 and 3 innate lymphoid cells in control and ATG treated animals. In all experiments, data from both groups were examined for normal distribution. Differences were statistically analyzed by unpaired t- or Mann-Whitney-test, depending on distribution. Bar graphs show the respective means ± SEM. n = 4–8 animals/group, respectively.

To identify major differences induced by treatment, we first followed an unbiased analysis approach employing the t-SNE (t-Distributed Stochastic Neighbor Embedding) algorithm embedded in FlowJo. For that, FACS data derived from organs of controls and ATG treated animals were tagged according to sample type, followed by concatenation into one single data file. Thereafter, t-SNE analysis was conducted, allowing unbiased assessment of dominant differences. Overlay of t-SNE plots for each organ type consistently identified clusters present in controls, but absent in treated animals (Figure 1B, left column). For identification of cell types driving this different clustering, expression levels of all markers used for t-SNE were overlaid, pointing to a reduction of CD4^+^ and CD8^+^ T cells in all organs, as exemplarily depicted in Figure 2B (right columns).

We further analyzed all datasets after manual gating (depicted in Supplementary Figure S2A), enabling quantification and statistical examination of differences as illustrated in Figure 2A, where frequencies of the indicated cell types are presented as percentage of the total CD45^+^ leukocyte population across all organs. Overall, lymphocytes were strongly diminished by ATG treatment, whereas this effect was not observed for myeloid cell subsets. In detail, CD4^+^ and CD8^+^ T cells were significantly reduced in lymphoid organs (lymph nodes and spleen) and almost completely depleted in blood, lung and kidney. With respect to the latter, raw data presented in Supplementary Figure S2B allows a more direct estimation of renal T cell numbers remaining after ATG administration.

NK cells showed a similar pattern with the exception of lymph nodes where NK cells were already rare in controls. Interestingly, B cells, identified according to B220 expression, showed a relative increase within the CD45^+^ leukocyte population in both lymphoid organs. Within the myeloid cell compartment, we observed only few changes following ATG injection. Of note, most prominent alterations were confined to the kidney and encompassed a moderate relative increase in frequencies of CD11c^+^MHC-II^+^ dendritic cells and CD11b^+^F4/80^+^ macrophages. In all other organs, Ly6C^+^MHC-II^-^ monocytes instead of macrophages were quantified as the dominating population that was only modestly, but significantly increased in blood of ATG treated animals.

Provided that lymphocytes represented the cell population most strongly affected by ATG treatment, we further aimed at analyzing innate lymphoid cells (ILCs), a multifaceted group of lymphocytes that does not express characteristic T-, B- or dendritic cell associated lineage markers [20]. Their transcriptional hallmarks broadly mirror the discrimination among T helper (Th) cell type 1, 2 and 17 subsets; accordingly, group 1 ILC, that include conventional NK cells, express T-bet, group 2 ILC are GATA-3^+^, whereas group 3 members are RORγt^+^. ILC type 2 cells have already been demonstrated to protect murine kidneys from glomerulosclerosis [21] and renal ischemia reperfusion injury [22]. Given the potential importance of ILCs in transplantation, we therefore particularly focused on the kidney for ILC analysis. The flow cytometric marker panel for ILC subset identification is depicted in Supplementary Figure S3 with the gating strategy being summarized in Supplementary Figure S4. Of note, ILC frequencies were calculated within CD45^+^lineage^−^ cells; therefore, they could not directly be compared to the leukocyte percentages depicted in Figure 2A. Following ATG treatment, we detected a significant reduction in ILC type 1 and 2 subsets compared to controls, whereas portions of the ILC3 subpopulation were not affected (Figure 2B).

As a summary, all major lymphocyte subpopulations with the exception of B cells and ILC3 were significantly diminished as a consequence of ATG treatment in both non-lymphoid (kidney, lung) and lymphoid (lymph node, spleen) organs as well as in peripheral blood.

### Impact of Donor ATG Pre-Treatment on Allogeneic Kidney Transplantation Outcome

**FIGURE 3:**
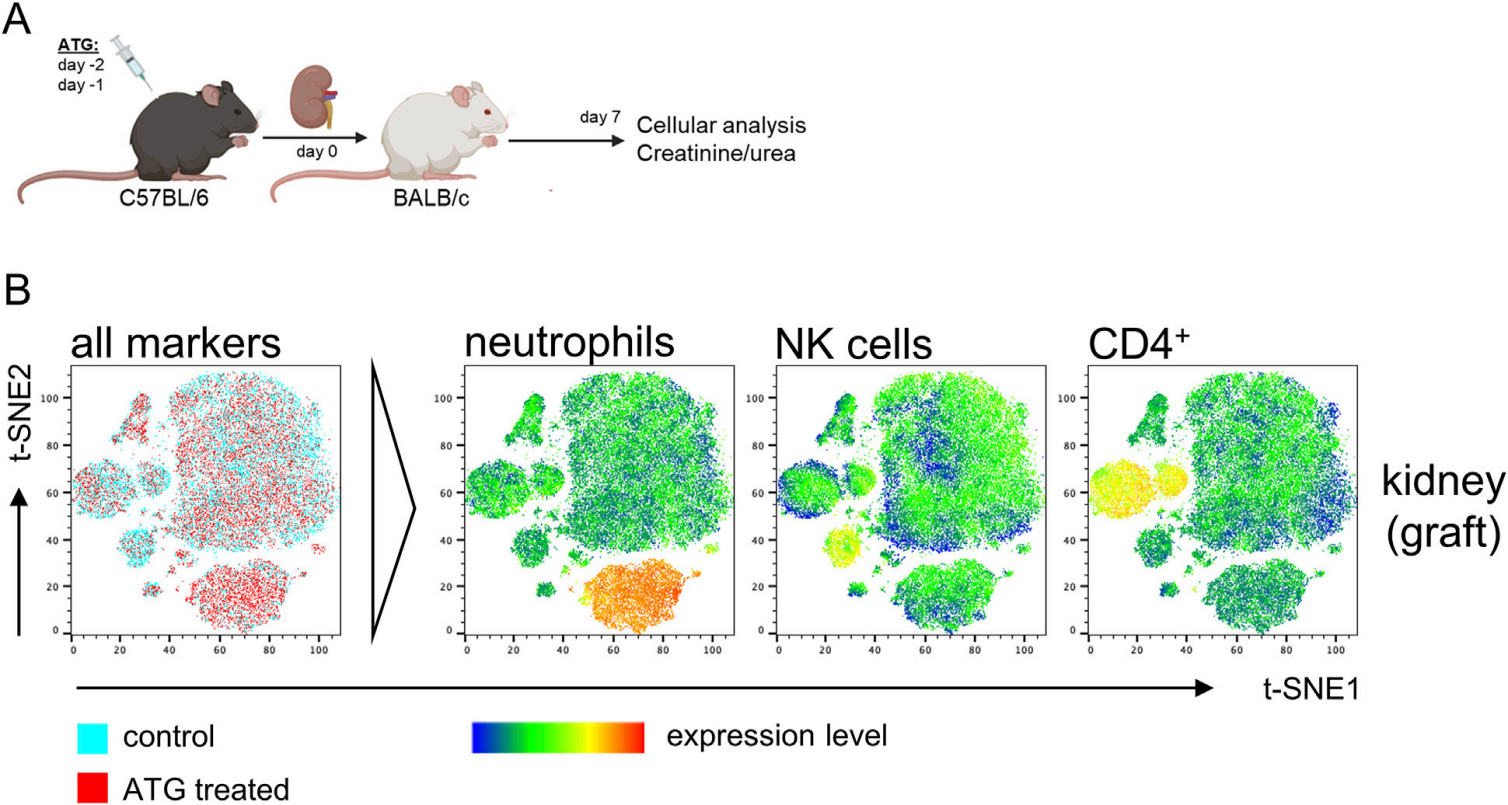
Impact of ATG donor-pretreatment on leukocyte composition in allografts after experimental kidney transplantation. **(A)** Experimental design and strain combination for murine kidney transplantation in context with intraperitoneal ATG pretreatment of the donor. **(B)** Exemplary t-SNE plots of flow cytometric data derived from kidney graft samples using the antibody panel shown in Supplementary Figure S1. For t-SNE analysis, renal cells were pre-gated on live CD45^+^ leukocytes; thereafter, datasets from n = 4–7 animals/group were concatenated. All remaining markers listed in Supplementary Figure S1 were used for clustering. Data from control (blue) and ATG-treated (red) animals were overlaid to identify differences in cluster composition. For prominently different clusters, cellular identity was determined based on expression levels of the respective lineage markers, thereby e.g., identifying increased neutrophil and decreased CD4^+^ T cell frequencies in kidneys from ATG-treated animals.

The persistence of donor-derived, intra-graft leukocytes after transplantation has been already shown to impact transplant survival and -function in clinical studies as well as in experimental models [8, 23]. We therefore addressed whether depletion of donor cells by ATG affects allogenic kidney transplantation outcome both on the cellular and functional level. As depicted in Figure 3A, kidneys from control or treated C57BL/6 mice were transplanted into BALB/c mice, representing a fully MHC-mismatched donor:recipient combination. Cellular and functional readout was performed on day 7, representing a typical timepoint in this acute rejection model, as recently published by us [5].

In an identical approach as for the analysis of ATG-induced alterations in naïve mice, we employed t-SNE for unbiased identification of pre-treatment effects on kidney transplantation outcome. For that, MNCs from the kidney graft were surface stained as depicted in Supplementary Figure S1 and data was acquired by FACS. Overlay of t-SNE plots indicated only few dominant differences in cellular composition (Figure 3B, left column). For identification of cell types driving slightly different clustering, expression levels of candidate markers were overlaid, pointing to a moderate reduction of graft CD4^+^ T and NK cells and an increase in neutrophils (Figure 3B, right columns).

**FIGURE 4:**
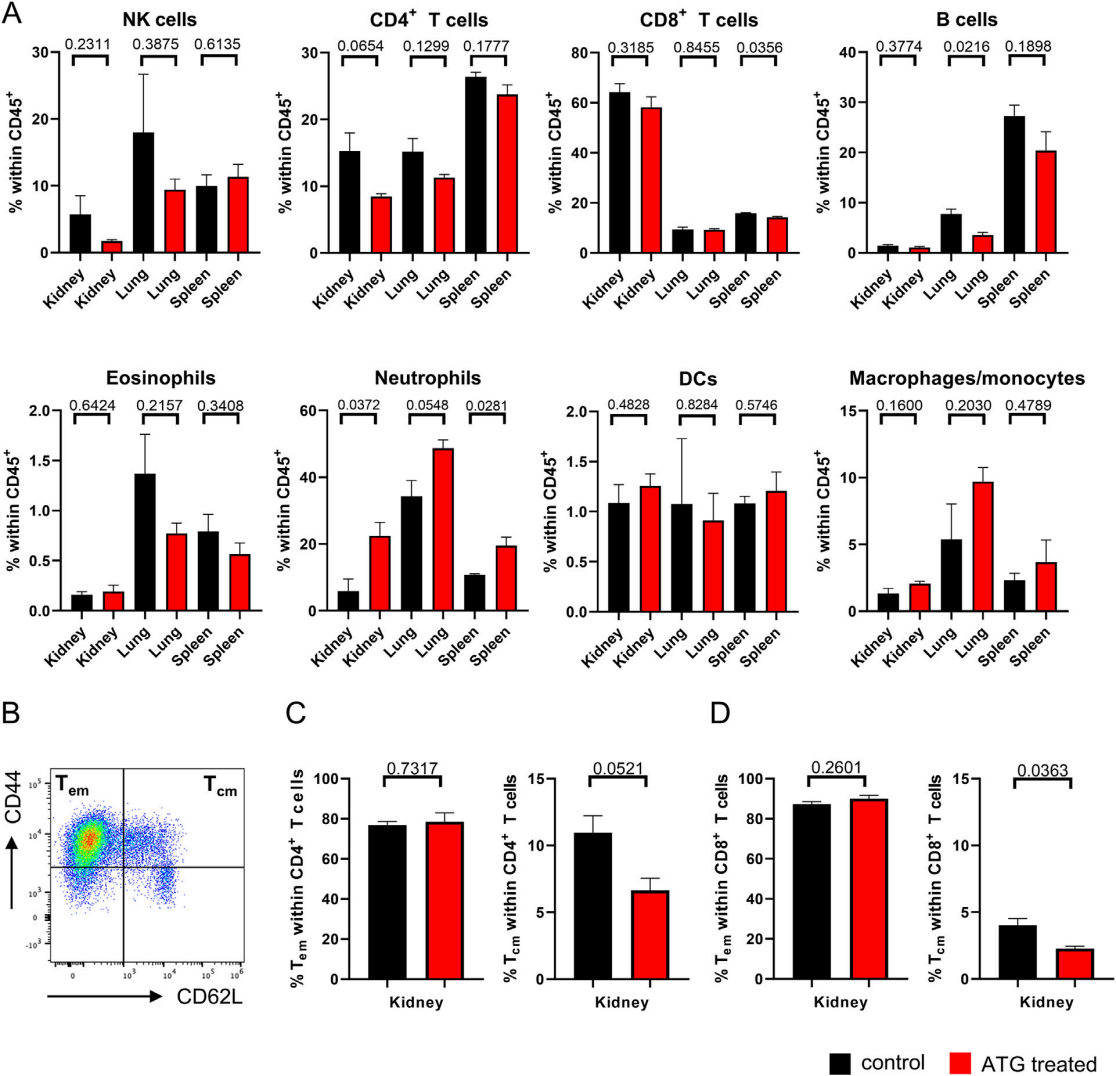
Organ-specific leukocyte composition after kidney transplantation. **(A)** The indicated leukocyte lineages were quantified by FACS in kidney, lung and spleen in mice receiving allogenic kidney grafts from either control or ATG-pretreated donors. **(B)** Definition of T_em_ and T_cm_ subsets based on the indicated marker combinations. Subset composition in renal **(C)** CD4^+^ and **(D)** CD8^+^ T cells from mice receiving allogenic kidney grafts of either control and ATG-pretreated donors. In all experiments, data from both groups were examined for normal distribution. Differences were statistically analyzed by unpaired t-oder Mann-Whitney-test, depending on distribution. Bar graphs show the respective means ± SEM. n = 4–7 animals/group.

We further analyzed all datasets after manual gating (Supplementary Figure S2), enabling quantification and statistical examination of differences as illustrated in Figure 4A. Of note, we did not detect additional intra-graft differences than those identified by t-SNE that did only reach statistical significance for neutrophils and a trend for CD4^+^ T cells. Analysis of MNCs from other organs showed a slight, but significant decrease in lung B cells in case of ATG donor-pre-treatment, whereas neutrophils were increased in spleen and showed a trend in lung.

Based on the finding that donor ATG-pretreatment resulted in moderately diminished frequencies of intra-graft CD4^+^ T cells, we conducted subanalyses, assessing quantities of effector-memory (T_em_) and central-memory- (T_cm_) type T cells identified according to CD44 and CD62L expression (Figure 4B). Interestingly, we noted a trend towards reduced portions of intra-graft CD4^+^ T_cm_ after ATG pre-treatment, whereas T_em_ remained unchanged (Figure 4C). The same applied to the CD8^+^ T cell compartment where the drop in T_cm_ frequencies reached significance (Figure 4D).

Importantly, as already demonstrated earlier using mice expressing the congenic markers CD45.1 and CD45.2 for donor/recipient discrimination [5], CD45^+^ donor leukocytes were gradually depleted in untreated animals until day 7 in our fully mismatched transplantation model, highlighting that more than 99% of analyzed cells at this timepoint were recipient-derived (Supplementary Figure S5A). Subset analysis revealed that a considerable number of renal TCRβ^+^ donor T cells is still detectable at day 3 post transplantation (Supplementary Figure S5B).

**FIGURE 5:**
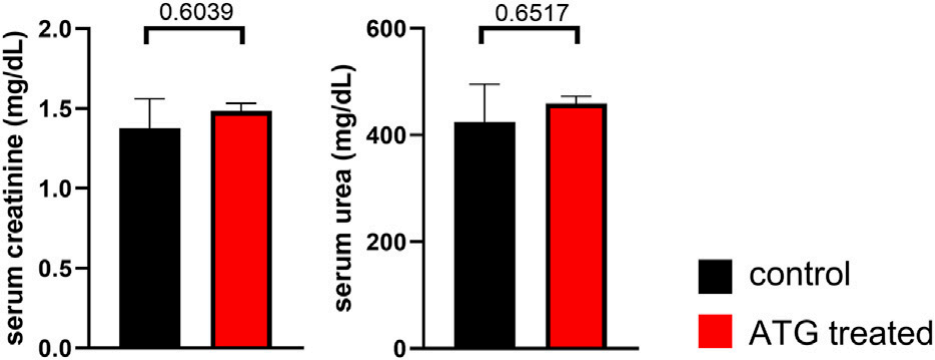
Impact of ATG treatment on allograft function. Serum creatinine and urea levels were determined on day 7 after allogenic kidney transplantation in mice receiving grafts of either control or ATG-pretreated donors. Data from both groups were examined for normal distribution. Differences were statistically analyzed by unpaired t-oder Mann-Whitney-test, depending on distribution. Bar graphs show the respective means ± SEM. n = 4–7 animals/group.

For deciphering the consequences of donor ATG-pretreatment on renal function, creatinine and urea levels were determined in serum on day 7 after transplantation. Importantly, we did not detect significant differences between graft recipients of control or ATG pre-treated donor organs (Figure 5).

In summary, ATG pre-treatment of the organ donor did only result in a minor cellular re-composition of the allograft in our transplantation model. In line with the aforementioned, we did not note relevant changes in graft function in association with this type of therapeutical intervention.

## Discussion

In this study, we experimentally followed the hypothesis that pre-treatment of a potential murine renal organ donor with ATG leads to changes in the immunological composition of the kidney, thereby potentially impacting the recipient´s allogenic immune response once the modified organ is transplanted. Such approach might be of particular interest in a setting where inferior long-term transplantation outcomes are to be expected, e.g., due to marginal organ usage from donors with advanced age or certain comorbidities (summarized in [10, 11]).

So far, it remained largely obscure how systemic administration of ATG particularly affects tissue-residing leukocytes in solid organs that might critically impact transplantation outcomes [24]. We chose a comparably high ATG dose of 25 mg/kg that had been previously demonstrated to reduce e.g., splenic T cells in naïve mice by up to 95% [25, 26]. Of note, a similar dosing (30 mg/kg) is clinically applied for immune ablation in severe autoimmunity before autologous stem cell transplantation [27]. For prevention of organ rejection in clinical kidney transplantation, our dosage exceeds commonly applied drug levels (typically 1.5 mg/kg Thymoglobuline^®^ [28]) where immunosuppression in the recipient has to be balanced with preservation of protective immunity. The latter considerations, however, do not apply in our organ donor-centered approach aiming at robust leukocyte depletion.

In our hands, as presented here, i.p. ATG treatment of naïve mice resulted in a strong and significant depletion of T-, NK- and innate lymphoid cells in both lymphoid and non-lymphoid organs, including the kidney. Of note, in accordance with *in vitro* data on anti-mouse ATG binding affinities to potential murine target cell populations [25], treatment spared B cells and all myeloid subsets. Particularly the latter aspect is of interest in the transplantation context, provided that donor-derived myeloid cells could contribute to allo-immunity by different mechanisms. These might not only involve direct presentation of donor antigens to recipient T cells (summarized in [29]), but also include the recently discovered priming of recipient by donor myeloid cells via CD47/SIRPalpha-polymorphisms [30]. Although these mechanisms argue in favor of myeloid cell depletion strategies in the donor, pointing to a potential limitation of our approach, their relevance needs to be critically examined within the time frame of the renal persistence of donor leukocytes, which we determined to fade until day 7 post transplantation in our model.

Despite efficient depletion of donor lymphocytes, we did not observe a pronouncedly altered cellular infiltration into the kidney after transplantation, nor an improvement of graft function.

In conclusion, the broad donor lymphocyte depletion approach chosen herein obviously does not substantially impact the course of alloimmune inflammation in the acute transplantation model. One explanation might be linked to our finding that donor-derived leukocytes are anyway rapidly depleted within 7 days after transplantation in such complete MHC mismatch setting [5] in the absence of immunosuppressive therapy. Therefore, an impact of so called “passenger leukocytes” might be little, given the specific framework of our experimental model.

The idea that donor-derived passenger leukocytes, being transferred to the transplant recipient, in turn influence anti-donor immunity dates back to the 1950ies [6]. In the meantime, several pre-clinical and clinical observations support a concept that certain transplant derived immune cell populations persist in the recipient and might, depending on the type of cells, contribute to desired or undesired outcomes. In that context, passenger donor CD4^+^ T cells have been shown to augment early antibody-mediated allo-immunity in a murine heart transplantation model by activating recipient B cells [8, 31]. Since this mechanism boosted allograft vasculopathy and early graft failure, it was concluded that “…passenger donor lymphocytes may therefore … represent a therapeutic target in solid organ transplantation” [8]. Importantly, the report elegantly revealed at the same time that the impact of donor lymphocytes on transplantation outcome is critically dependent on the degree of donor:recipient mismatch; in a fully mismatched heart model, recipients’ NK cells are rapidly depleting donor T cells, thereby preventing an effect on alloimmunity [8]. Whether and how such mechanism of donor T cell help to recipient B cell activation might contribute to donor specific antibody (DSA) production in the murine kidney transplantation model is a complex question that is out of the scope of our small study. At least with respect to donor T cell persistence in the first days after transplantation (according to our data, at least until day 3), we cannot principally exclude it, also considering that we isolated comparable numbers of donor T cells from kidney grafts as those recovered from hearts in the report by Charmetant [31]. Furthermore, DSA have been detected in rat and murine kidney transplantation models from day 5–7 post transplantation, supporting a critical role for very early T:B cell interactions [32, 33].

Conversely, it cannot be excluded that certain T cell subsets being transferred with the graft might serve desired (that is, anti-inflammatory) functions. In this regard, cardiac graft survival was found to be positively associated with the presence of natural CD4^+^ regulatory T cells, provided that their depletion pre-transplantation augmented alloimmunity [9]. Similar associations were demonstrated in clinical and murine lung transplantation, where tissue-resident Tregs control humoral rejection [7]. In humans, a subset of donor-derived T lymphocytes, termed tissue-resident memory T cells (T_rm_), being present in transplanted lungs, has recently been found associated with beneficial clinical outcomes. The fact that these cells were predominantly and long-term detectable in bronchoalveolar fluid, but not in peripheral blood, underscores the need for analyzing alloimmunity directly in target tissues [23].

In conclusion, the aforementioned reports point to a main limitation of our approach in that depletion of donor leukocytes pre-transplantation might be a double-edged sword and that a more celltype-selective strategy, e.g., by sparing graft-resident, donor-derived regulatory or resident memory-type T cells, should be envisioned. This might also be accomplished by titrating ATG dosage, provided that reduced drug levels could have slightly different effects on T cell subsets, as demonstrated in human kidney transplant recipients [34]. Major limitations also include that we did not particularly assess alloimmunity after transplantation; it cannot be excluded that subtile ATG treatment effects are only detectable at the antigen-specific level, suggesting that future studies should include examination of allo-specific cellular and/or humoral responses. Indeed, with respect to the latter, the report by Charmetant et al. [31] highlighted that a few thousand donor-derived T helper cells might suffice to prime donor-specific antibody production in the heart model.

Furthermore, the translational potential of our findings might be limited due to differences between targets of anti-murine ATG and the respective human drug(s): Whereas we did not document altered B cell frequencies in naïve mice after ATG treatment, anti-human Thymoglobuline (Sanofi) has been demonstrated to deplete CD19^+^ B cells in a humanized mouse model [35]. The fact that this observation could not be reproduced after ATG administration to kidney transplant recipients [36] highlights that the study design should be critically scrutinized in view of its clinical transferability. In that context, human kidney transplantation usually involves selection of the donor organ according to MHC-matching and requires life-long administration of immunosuppressive drugs. Both aspects likely shape maintainance and functionality of passenger leukocytes in the clinical setting. The future challenge will be to better mirror these features in an adapted experimental model that will also consider the impact of passenger leukocytes in the chronic phase of anti-donor immunity.

## Data Availability

The original contributions presented in the study are included in the article/Supplementary Material, further inquiries can be directed to the corresponding authors.
