# Supplementary material for: Impact of Organ Donor Pretreatment With Anti-Thymocyte Globulin in a Murine Model of Allogenic Kidney Transplantation
Source: Transpl Int. 2025 Jan 7;37:13997. doi: 10.3389/ti.2024.13997 (PMC11745874; doi:10.3389/ti.2024.13997)
Supplement: Supplementary file 1 [file DataSheet1.PDF]

Supplemental Figure 1

#	Cell type	Molecule	Fluorochrome
1	Live cells	Viability dye	BV510
2	NK cells	Nkp46	Alexa Fluor 647
3	T	CD3	PerCP-Vio700
4	Th	CD4	BV711
5	Tc	CD8	BV605
6	B	B220	BV650
7	DCs	CD11c	PE
8	Mono	CD11b	BV785
9	HLA	MHCII	BV421
10	Leukocytes	CD45	BUV395
11	Neutrophils	Ly6G	Pe-Cy7
12	Macrophages	F4/80	APC-Cy7
13	Mono	Ly6C	Pe-Dazzle594
14	Eosinophils	Siglec-F	Alexa 700

FACS panel for assessment of the main leukocytes lineages in murine tissues and blood

Supplemental Figure 2

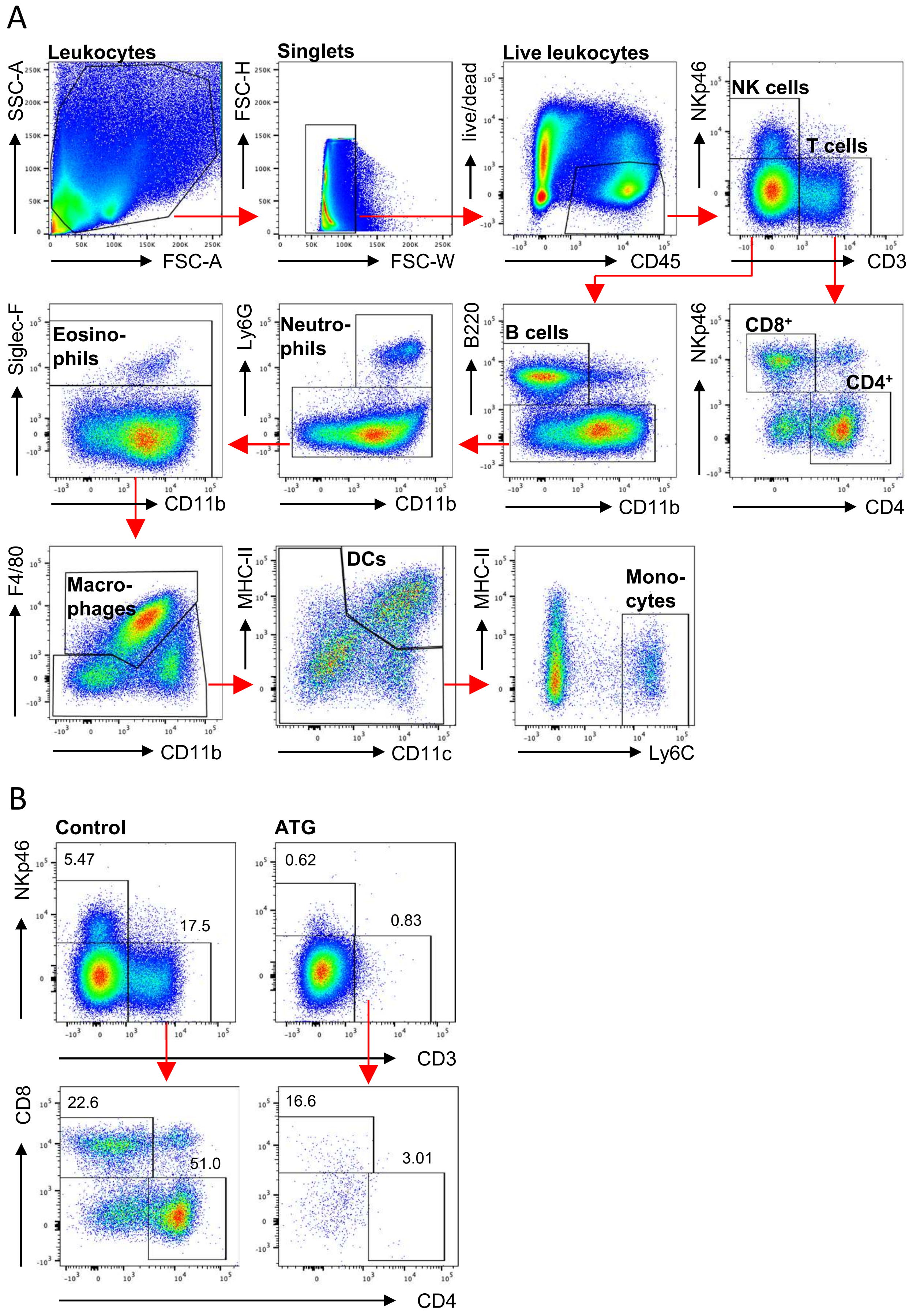

(A) Gating strategy for manual identification of the indicated cell lineages based on the FACS panel listed in Supplemental Figure 1; exemplarily depicted for kidney of a control animal. (B) Exemplary plots for the degree of T cell depletion from kidneys of naive animals treated or not with ATG; approx. 50% of all isolated renal cells were acquired by FACS and all acquired cells are depicted after gating on live leukocytes.

Supplemental Figure 3

#	Cell type	Molecule	Fluorochrome
1a	Lineage	B220	APCCy7
1b	Lineage	CD11c	APCCy7
1c	Lineage	GR1	APCCy7
1d	Lineage	F4/80	APCCy7
1e	Lineage	FcER1a	APCCy7
1f	Dead cells	L/D dye	APC-Cy7
2	Leukocytes	CD45	BV510
3a	T cells	TCRβ	PerCPCy5.5
3b	T cells	TCRγδ	PerCPCy5.5
4	ILCs	CD127	BV785
5	ILCs	NKp46	BUV737
6	ILCs	T-bet	Alexa647
7	ILCs	EOMES	Alexa488
8	ILCs	GATA3	PE
9	ILCs	RORγt	BV421

FACS panel for assessment of innate lymphoid cell subsets.

Supplemental Figure 4

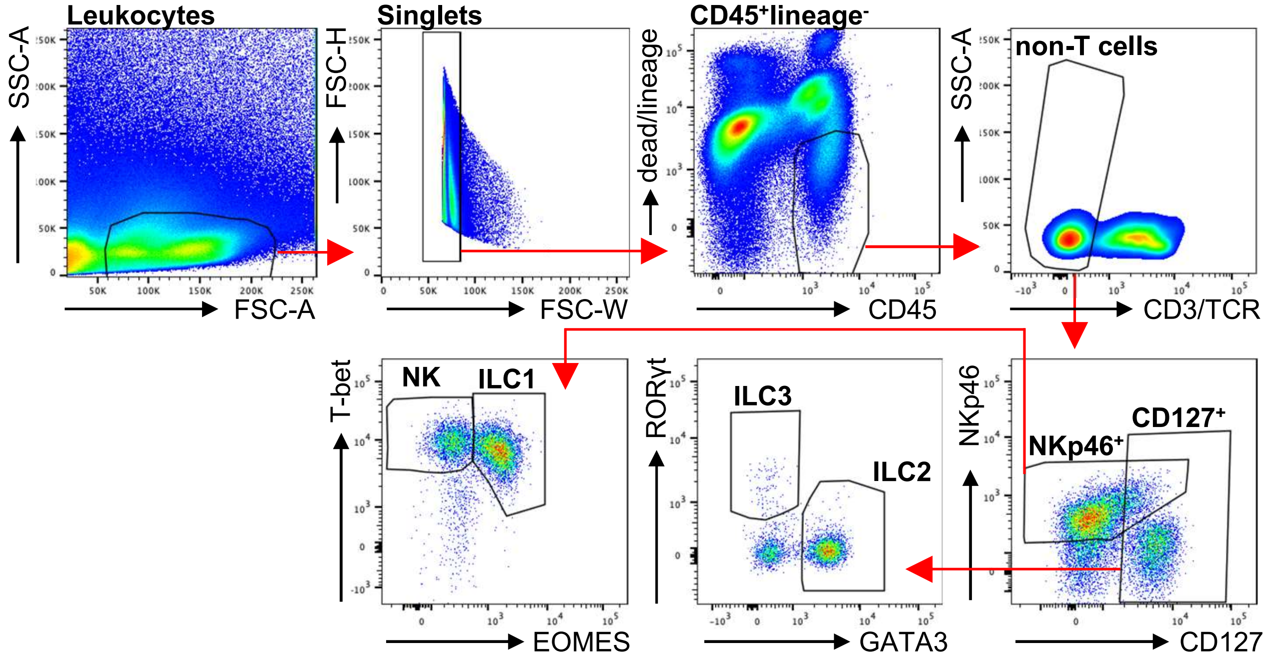

Gating strategy for manual identification of innate lymphoid cell subsets (NK cells and ILC type 1-3) according to transcription factor expression and based on the FACS panel listed in Supplemental Figure 3; exemplarily depicted for kidney of a control animal.

Supplemental Figure 5

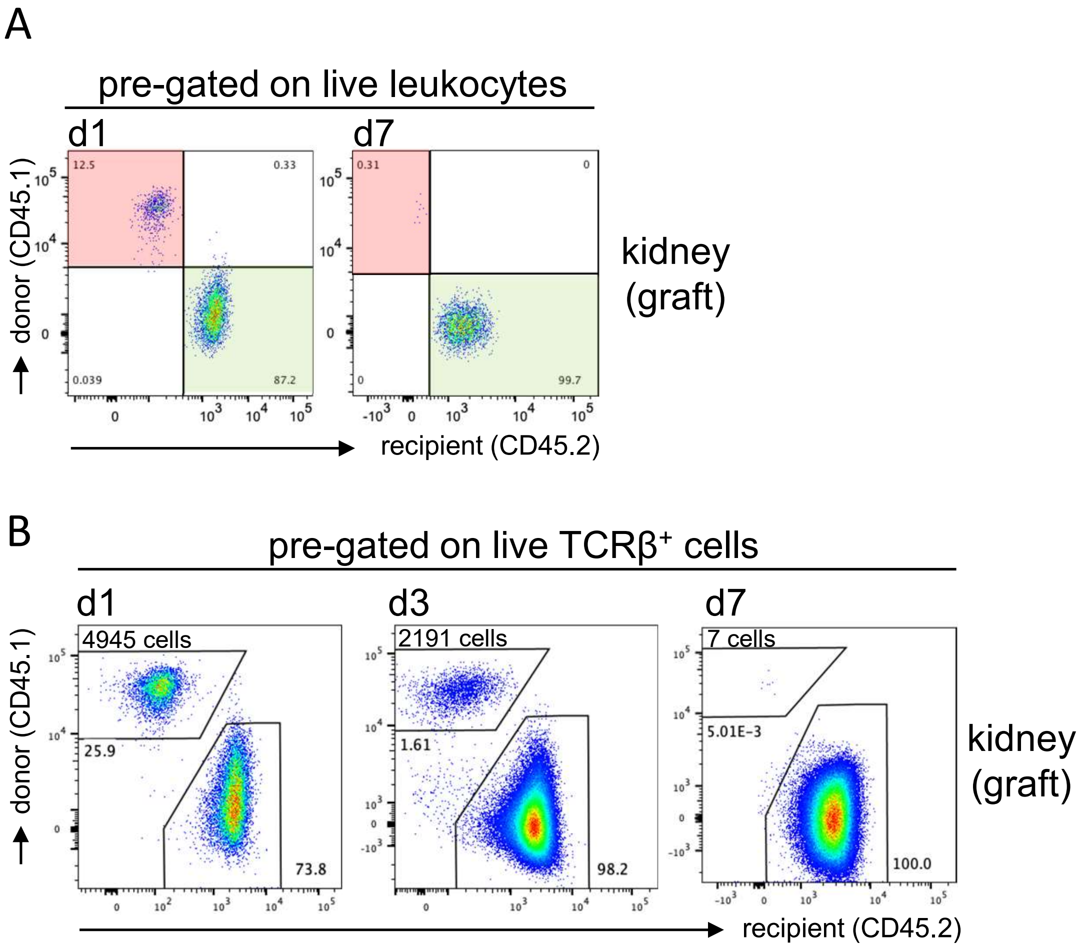

(A) Contribution of donor and recipient derived leukocytes to renal cell composition after kidney transplantation. Leukocyte composition in renal grafts was assessed on the indicated days after transplantation according to the congenic markers CD45.1 (donor) and CD45.2 (recipient) by FACS after pre-gating on live cells. (B) Kinetics of donor T cell persistence in the renal allograft at the indicated time points; approx. 50% of all isolated renal cells were acquired by FACS and are shown. Data depicted in (A) and (B) derive from different experiments.
